# Investigating #covidnurse Messages on TikTok: Descriptive Study

**DOI:** 10.2196/35274

**Published:** 2022-01-14

**Authors:** Bhavya Yalamanchili, Lorie Donelle, Leo-Felix Jurado, Joseph Fera, Corey H Basch

**Affiliations:** 1 William Paterson University Wayne, NJ United States; 2 Western University London, ON Canada; 3 Lehman College Bronx, NY United States

**Keywords:** COVID-19 pandemic, nurse, burnout, social media, stress, TikTok, nursing, COVID-19, pandemic, social support, digital peer support, health communication, peer support

## Abstract

**Background:**

During a time of high stress and decreased social interaction, nurses have turned to social media platforms like TikTok as an outlet for expression, entertainment, and communication.

**Objective:**

The purpose of this cross-sectional content analysis study is to describe the content of videos with the hashtag #covidnurse on TikTok, which included 100 videos in the English language.

**Methods:**

At the time of the study, this hashtag had 116.9 million views. Each video was coded for content-related to what nurses encountered and were feeling during the COVID-19 pandemic.

**Results:**

Combined, the 100 videos sampled received 47,056,700 views; 76,856 comments; and 5,996,676 likes. There were 4 content categories that appeared in a majority (>50) of the videos: 83 showed the individual as a nurse, 72 showed the individual in professional attire, 58 mentioned/suggested stress, 55 used music, and 53 mentioned/suggested frustration. Those that mentioned stress and those that mentioned frustration received less than 50% of the total views (n=21,726,800, 46.17% and n=16,326,300, 34.69%, respectively). Although not a majority, 49 of the 100 videos mentioned the importance of nursing. These videos garnered 37.41% (n=17,606,000) of the total views, 34.82% (n=26,759) of the total comments, and 23.85% (n=1,430,213) of the total likes. So, despite nearly half of the total videos mentioning how important nurses are, these videos received less than half of the total views, comments, and likes.

**Conclusions:**

Social media and increasingly video-related online messaging such as TikTok are important platforms for social networking, social support, entertainment, and education on diverse topics, including health in general and COVID-19 specifically. This presents an opportunity for future research to assess the utility of the TikTok platform for meaningful engagement and health communication on important public health issues.

## Introduction

Nurses play an integral role in health care, with around 28 million nurses worldwide. The nursing profession dominates the variety of occupations in the health care industry, with about 59% of professionals. Despite nurses accounting for the majority of health care professionals, there is still a need for 5.9 million nurses globally [1].

Due to the COVID-19 pandemic, there has been an overwhelming number of deaths including many health care workers. The International Council of Nurses (2020) confirms 1500 nurses perished from COVID-19 in 44 countries [2]. The nature of the nursing role creates situations where most nurses are at high risk of being overworked, experiencing burnout, and exposed to potentially psychologically traumatizing events [3]. This has only been exacerbated by the onset of the COVID-19 pandemic. Previous research conducted during similar outbreaks (H1N1, severe acute respiratory syndrome) showed that nurses experienced the highest levels of work-related stress, and subsequent distress, compared to other groups of health care providers [4].

The pandemic heightened and brought to public attention many health care–related disparities. Nurses encountered many new and difficult challenges resulting from stress. The rates of severe stress among nurses have been reported as high as 56.4% [5]. The proportion of mental health issues among nurses during the pandemic are markedly higher than the general population prevalence of anxiety and depression estimated at 11.2% and 4.7%, respectively [6].

One of the most prominent causes of stress among nurses during the COVID-19 pandemic is the persistent fear of infection due to their proximity to those already ill. Contracting the infection as well as the possibility of spreading it to loved ones is reported as a major fear of nurses, thereby increasing stress levels both on the job and at home [7]. Many nurses were unable to stay with their loved ones during the beginning of the pandemic, elevating the potential for chronic stress, grief, and anxiety [8]. One study that looked specifically at nurses living in isolation to protect their relatives found high rates of stress in 39.1% of the participants [9].

Working long hours at the frontlines of the COVID-19 pandemic, nurses were substantially impacted by the lack of resources, increased physical and emotional stress, and increased risk of harm [10]. The lack of resources proved to be a substantial stressor for nurses, especially during the initial months of the pandemic when adequate personal protective equipment (PPE) was unavailable. Anecdotal accounts from the beginning of the pandemic consistently showed that concerns regarding inadequate PPE increased symptoms of anxiety, stress, and fear among nurses and other health care workers [3,11]. Shahrour and Dardas [7] found that the lack of PPE was associated with higher stress and worse mental health outcomes. Moreover, hospitals were inundated with COVID-19 admissions, as a result, nurses were given little reprieve from the high workload and the uncertainty of when the pandemic will end [8]. Global mental health experts note that COVID-19–related challenges increased the risk of moral harm and emotional stress [12]. Nurses providing patient care during unprecedented times and complex decision-making, and repeatedly seeing traumatic events resulted in anxiety, depression, exhaustion, and burnout [12,13].

Over the past 15 years, social media use has increased substantially, with around 72% of the American public using some type of social media platform [14]. These platforms are available for individuals of all genders and backgrounds; user demographics of these platforms vary as they garner different audiences. Research indicates that 78% of US adult women use at least one type of social media platform like TikTok or YouTube, compared to 66% of US adult males [14].

TikTok essentially functions as a repository of short-form mobile videos [15]. The social media app is marketed as a platform to develop creativity and content development skills, an edutainment portal, and a vehicle for commercial advertising [16]. TikTok, a user-friendly platform originally designed to engage young audiences, enables users to develop, create, and share content and campaigns to a large and diverse audience. An influencer is an emerging role on social media defined as “everyday, ordinary Internet users who accumulate a relatively large following on blogs and social media through the textual and visual narration of their personal lives and lifestyles, engage with their following in digital and physical spaces, and monetize their following by integrating ‘advertorials’ into their blog or social media posts” [17]. Although nurses do not engage in commercial marketing, health communication can also be viewed under the umbrella of social marketing [18]. Nurses require knowledge and communication skills to reach the growing number of individuals and families who access health and health-related information through online and social media channels. However, the use of social media by health care professionals is a contentious issue; the presence of online misinformation challenges the integrity of online health information, and although many providers are embracing social media use, others perceive its use as unprofessional [19].

Social media in the nursing workplace can be beneficial if it is used appropriately. Social media has shown to enhance professional networking and provide an outlet to share feelings and seek support from other nurses and health care professionals. It also serves as a way to inform and educate consumers and colleagues, and can even enhance timely communication with patients and members of the health care team [20]. During a time of high stress and decreased social interaction, nurses have turned to social media platforms like TikTok as an outlet for expression, entertainment, and communication. Anecdotally, nurses have used #TiredHealthcareWorker on TikTok to document and expose untenable COVID-19–related working conditions, the unrelenting stress, and the impact on their mental health [21]. There is a clear lack of research published in this area. In response to this gap in the research literature, the purpose of this cross-sectional study was to describe the content of videos on the hashtag #covidnurse on TikTok.

## Methods

This cross-sectional content analysis study included 100 videos in the English language from the hashtag #covidnurse on TikTok. At the time of the study, the hashtag had 116.9 million views. The videos were excluded if they were not in the English language and were not relevant to the subject being explored, nursing and COVID-19. The coding categories were finalized on July 19, 2021, whereas the coding was completed on August 14, 2021. The videos were included if they were in the English language and were relevant to the subject being explored, both nursing and COVID-19. The first 100 videos using the search term #covidnurse to have these criteria were included. For each video, the date of posting, number of views, comments, and likes were indicated. All videos were also analyzed for mention or suggestion of the content categories, which were created inductively and collaboratively. Data was collected by observing TikTok videos for mention or suggestion of various predetermined categories. Additionally, information about the creators was noted based on the video, bio, or caption. All the data from the video were collected, categorized, and organized using Excel (Microsoft Corporation). A single reviewer (BY) watched all 100 videos and indicated whether or not the content categories were present in each video. A second reviewer (CHB) watched 10 (10% of the sample) randomly selected videos for the same content to determine interrater reliability. Of the 220 data points, the two reviewers only differed on 3 points, resulting in a score of k=0.97.

Organization, data entry, and analysis were all completed in Excel. Descriptive statistics and independent 1-tailed *t* tests (*α*=.05) were completed to determine if the predetermined content characteristics had an impact on video interaction based on views (number of times the video was watched), likes (number of times the like button was hit indicating a level of approval), or comments (number of individual remarks) a video garnered. This study was exempt from review, as the William Paterson University Institutional Review Board does not review methods that do not involve human participants.

## Results

Combined, the 100 videos sampled received 47,056,700 views; 76,856 comments; and 5,996,676 likes. The respective averages were as follows: 470,567 (SD 1,232,554.2); 769 (SD 1626.44); and 59,967 (SD 231,319.51). Of the 100 videos, 39 mentioned safety, 54 did not mention safety, and 7 mentioned nurse-to-patient ratios. Table 1 shows different content characteristics and indicates how many of the videos reviewed included this content. The number of views, comments, and likes for videos with this content is also given in the table. Note that two content categories were eliminated from the table since they were not observed in any of the videos sampled. They were “mentions suicide” and “has misinformation.”

**Table 1:** Observed content, views, comments, and likes of 100 TikTok videos on nursing.

	Videos (n=100), n	Views (n=47,056,700), n (%)	Comments (n=76,856), n (%)	Likes (n=5,996,676), n (%)
Individual is a nurse	83	43,894,700 (93.28)	69,398 (90.30)	5,662,290 (94.42)
Individual in professional attire	72	41,016,100 (87.16)	50,933 (66.27)	5,316,127 (88.65)
Mentions/suggests stress	58	21,726,800 (46.17)	45,405 (59.08)	2,562,825 (42.74)
Uses music	55	27,115,100 (57.62)	43,429 (56.51)	2,938,897 (49.01)
Mentions/suggests frustration	53	16,326,300 (34.69)	44,630 (58.07)	2,288,307 (38.16)
Mentions/suggests importance of nursing	49	17,606,000 (37.41)	26,759 (34.82)	1,430,213 (23.85)
Mentions/suggests social support	44	11,830,701 (25.14)	31,251 (40.66)	1,598,048 (26.65)
Uses humor	40	25,442,101 (54.07)	23,770 (30.93)	3,829,229 (63.86)
Mentions/suggests exhaustion	40	14,486,500 (30.79)	34,305 (44.64)	1,481,081 (24.70)
Mentions/suggests mental health	34	7,785,400 (16.54)	27,474 (35.75)	1,066,005 (17.78)
Using post as encouragement	32	10,716,500 (22.77)	15,457 (20.11)	963,531 (16.07)
Mentions/suggests being strong (mentally, physically, or emotionally)	26	11,613,400 (24.68)	24,872 (32.36)	1,449,092 (24.16)
Mentions/suggests being scared	25	7,507,000 (15.95)	26,791 (34.86)	1,164,559 (19.42)
Mentions/suggests hours worked	19	4,687,400 (9.96)	9590 (12.48)	408,386 (6.81)
Mentions/suggests burnout	19	1,089,400 (2.32)	6653 (8.66)	90,661 (1.51)
Mentions/suggests death (in relation to professional role regarding patient death or within the context of COVID-19–related deaths)	17	7,899,200 (16.79)	24,574 (31.97)	1,564,957 (26.10)
Uses dance	15	5,138,900 (10.92)	4685 (6.10)	361,124 (6.02)
Mentions/suggests COVID-19 vaccine	3	857,600 (1.82)	4082 (5.31)	81,800 (1.36)

There were 4 content categories that appeared in a majority (>50) of the videos: 83 showed the individual as a nurse, 72 showed the individual in professional attire, 58 mentioned/suggested stress, 55 used music, and 53 mentioned/suggested frustration. Despite the fact that videos with these content characteristics made up the majority, some did not garner a majority of the roughly 47 million total views. Those that mentioned stress and those that mentioned frustration received less than 50% of the total views (n=21,726,800, 46.17% and n=16,326,300, 34.69%, respectively). These observations, though, were not statistically significant. Independent 1-tailed *t* tests (*α*=.05) were run to see if videos with this content garnered significantly more views, comments, and likes than videos without this content. All tests resulted in *P* values larger than .05. Hence, it does not appear that mentioning stress or mentioning frustration resulted in significantly more views, comments, or likes. Although not a majority, 49 of the 100 videos mentioned the importance of nursing. These videos garnered 37.41% (n=17,606,000) of the total views, 34.82% (n=26,759) of the total comments, and 23.85% (n=1,430,213) of the total likes. So, despite nearly half of the total videos mentioning how important nurses are, these videos received less than half of the total views, comments, and likes. These observations, however, were also not statistically significant as determined by independent 1-tailed *t* tests (*α*=.05). The respective *P* values were .19, .18, and .095.

## Discussion

Although the year 2020 will be best remembered for the COVID-19 pandemic, 2020 was also distinguished globally as the year of the nurse and midwife. Over the course of the pandemic, the tireless work of nurses has been highly regarded in the mass media and through public displays of appreciation worldwide. Nurses have been indispensable to mitigating and containing the spread of COVID-19. However, their strong sense of duty to patients, their families, and their nursing colleagues was not without personal consequences to their physical and emotional well-being [10,22]. This research assessed the TikTok video messages conveyed within the hashtag #covidnurse.

TikTok videos in this study focused primarily on nurses’ health and well-being, acknowledging the challenges faced by nurses, and provided a number of supportive and encouraging messages. Interestingly, messages related to COVID-19 and related to health promotion actions were only briefly mentioned. This finding seems inconsistent with others who have reported the importance of websites and social media as a health information resource during the pandemic [23]. Even more compelling is the notion that visual or illustrated health messages posted on platforms such as TikTok can reach and impact audiences with diverse health and digital health literacy skills; visual messaging can help to engage *hard to reach* people, contributing to information equity [24]. In our study, there were limited mentions of COVID-19 vaccines and no mention of misinformation. Yet, the World Health Organization has positioned misinformation (particularly online information) as a parallel pandemic and a substantial barrier to an efficient resolution to the COVID-19 outbreak. It may be the #covidnurse term emphasized nurses’ experiences in providing direct care to patients with COVID-19 in acute care settings rather than nurses’ role as health educator and communicator.

This study is limited by the inclusion of only one hashtag, studying content in English only, and the cross-sectional approach in which data was collected. Collecting data from videos only in English is limiting because neither the COVID-19 pandemic nor nursing care are geographically limited. Another limitation of the study is the fact that the data represents a snapshot in time and the results cannot be generalized. As the pandemic and social media evolve, the content from this hashtag also has the potential to change. However, this is the best possible assessment of the content studied as of the study closing date. Further, the presence of bots on this study sample, although highly unexpected, could not be verified. Despite these limitations, this study serves as an example of the type of content in this understudied area of research.

Social media and increasingly video-related online messaging such as TikTok are important platforms for social networking, social support, entertainment, and education on diverse health and related topics including COVID-19 [25-27]. In a study assessing nursing students’ COVID-19–related tweets, researchers reported that tweets “...praised the hard work of the medical staff, and urged the public to take responsible actions [compliance with public health recommendations]” [28]. In their systematic review of acute care nurses’ pandemic work experiences, researchers found a high level of collegial support among nurses during the pandemic and expressed concern among practicing nursing about caring for and about the health and well-being of their coworkers [22]. This is consistent with the findings from this study where we report a high proportion of videos that highlight stress, frustration, exhaustion, and mental health issues relevant to nursing within the context of COVID-19. The TikTok hashtag #covidnurse may serve as a platform for nurses and others to process their experiences, give and receive empathy, and lighten their emotional load through sharing with others, bearing witness in the virtual sense [29].

In addition, several messages contained within #covidnurse also reflect compassionate messages of humor, peer support, and remaining strong [22]. Godfrey and Scott [30] found that nurses engaged in a peer support program during the COVID-19 pandemic benefitted from reduced stress, increased resilience, and a sense of community. The TikTok #covidnurse online site may provide nurses an *always available* informal peer support environment where viewers will see they are not alone, are appreciated for their work, experience empathy, and have their thoughts and feelings validated by their peers [30].

Despite the supportive video messages reported within this study, there is a growing concern regarding malicious and hurtful feedback from TikTok viewers. These vitriol responses are a growing concern across online social media platforms [31]. Comments in this study were not analyzed according to the civility of the comments; however, nursing students’ tweets about the COVID-19 pandemic “used inappropriate language such as profanity and name-calling on this public platform.” Similar findings were noted in a 2019 study of uncivil tweets among nursing students and nurses [28]. This presents an opportunity for future research: to assess the civility of TikTok commentary and therefore the utility of the TikTok platform for meaningful engagement and health communication on important public health issues. TikTok and other social media platforms are increasingly important health promotion and health communication venues with considerable audience impact. TikTok is an especially relevant platform for health promotion and health education targeted to children and young adults, presenting health information in a way that is engaging and relevant to a substantial online audience. The peer-to-peer nature of TikTok also serves as an accessible source of health information, social support, and health promotion coaching among the users [32]. Nurses, perceived as trusted health care providers, may find TikTok to be an especially important vehicle for health promotion activities for children and young adults. Further this research indicates the ability of TikTok to serve as a catalyst for social support. In conclusion, this research has important implications for nursing practice and the use of ethical and effective social media platforms for health promotion.

## Abbreviations

PPE: personal protective equipment
